# Improving value for underserved populations with a community-based intervention: a retrospective cohort study

**DOI:** 10.1186/s13690-023-01117-z

**Published:** 2023-05-29

**Authors:** Claude Pinnock, John Rothen, Tom Carlough, Nirav R. Shah

**Affiliations:** 1 Wider Circle, Redwood, CA USA; 2 Clinical Excellence Research Center, Stanford University, Stanford, CA USA

**Keywords:** Social Determinants of Health, Inequity, Community based health, Closing gaps, Member outcomes, Lower costs, Higher value

## Abstract

**Background:**

Healthcare inequity drives high costs, worse outcomes and is heavily influenced by social determinants of health (SDOH). Addressing health behaviors and SDOH through a culturally competent community-based exposure may be effective in improving value for Medicaid enrollees. This study aims to evaluate whether such an exposure lowers costs at equal or improved quality.

**Methods:**

A retrospective cohort study leveraging claims data was conducted in Detroit, Michigan from April 2021 to April 2022 to examine the impact of a community-based peer support program on clinical, utilization and financial outcomes. A one-to-one propensity matching of 738 pairs of African American Medicaid enrollees was generated, and compared the difference of differences between inpatient, emergency department, prescription and outpatient paid amounts, utilization, and available claims-based quality metrics.

**Results:**

Compared to controls, peer support recipients generated significantly lower per member per month costs ($115, (95% CI $20.2 to $210)). Recipients showed a significant increase in the Adult Access to Preventative/Ambulatory Health Services 20–44 year old quality metric (8.31% (95% CI 0.35–16.3%)). Member retention in the health insurance plan was significantly higher for peer support recipients vs. the control group by 3.62% (p < 0.05). Peer support recipients displayed non-significant improvement on all other utilization and actuarial measures. No significant difference was found for any of the other examined quality metrics.

**Conclusions:**

Among a population of African American Medicaid enrollees, a culturally competent community-based intervention was associated with lower cost and better member retention with preserved or improved quality.

**Supplementary Information:**

The online version contains supplementary material available at 10.1186/s13690-023-01117-z.

**What is already known on this topic** – Health inequity and SDOH are well known to influence and drive low value care. It is unclear how effective supplemental benefits leveraging community to address these factors are, and how they differ for different ethnicities.

**What this study adds** – This study shows that an upstream community intervention may be an effective benefit to address inequity and SDOH for African American populations and lower costs whilst improving or maintain quality.

**How this study might affect research, practice, or policy** - Health payers, whether national or privatized, could look to contract with existing community-based wellness services to better manage risk and retain their populations, especially for members who are prone to healthcare disparities.

## Introduction

Healthcare in the United States accounted for 19.7% of gross domestic product (GDP) in 2020 [1]. The reasons for this are multifactorial and include an aging population, more expensive unit costs, and the recent COVID-19 pandemic [2, 3]. Patients who are insured by Medicaid represent $671.2 Billion US Dollars ($USD) of spend per year, approximately 16% of overall healthcare expenditures [4]. Historically underserved racial and ethnic groups account for 37% of the US population and 47% of Medicaid enrollees [5] and have worse outcomes and highest variation in access and cost when compared to their peers [6, 7].

While figures vary, social determinants of health (SDOH) are increasingly seen as an underlying driver of up to 30–80% of variation in health outcomes and costs [8]. SDOH are defined as “the conditions in the places where people live, learn, work, and play that affect a wide range of health and quality-of-life risks and outcomes” [9]. Improving SDOH was not traditionally in the purview of healthcare providers or payors, as the value could not be easily quantified or captured [10]. Today that remains the rule, with a few notable exceptions such as Kaiser Permanente providing supportive housing [11].

The Medicaid program was designed to provide health services to low income and disabled people and is therefore a relevant population for studying outcomes for historically underserved populations. These individuals often have difficulty accessing care including preventive health and must resort to using emergency care when disease is advanced [12]. A “perfect” storm is created: baseline inequity, a healthcare system not equipped to address SDOH, and poor access all drive worse health outcomes and higher costs for Medicaid members, especially among racial and ethnic minorities [13].

Improved health can be achieved by evidence-based interventions addressing SDOH in communities [14–17]. Unfortunately, such interventions are often delivered through fragmented services that are neither scalable nor sustainable. This study reports on a holistic, efficient, scalable, and community-based approach that uses a culturally competent team to improve health behaviors and SDOH.

Community engagement specialists in the Connect for Life (CFL) program form and manage peer groups in selected neighborhoods to create trusted peer-to-peer social connections, demonstrate the importance of preventative care, address SDOH and barriers to obtaining care, and improve care navigation and health behaviors through group-based dynamics [18, 19]. The peer-to-peer engagement forum uses the psychology of influence by allowing individuals to socially connect in comfortable ways with their peers and connect with credible information sources, while becoming more astute consumers of health services in their local community.

For African American Medicaid members, this study aimed to understand whether CFL:

- decreased total cost of care,
- reduced inpatient and non-emergent Emergency Department (ED) utilization,
- maintained or improved key clinical quality metrics such as cancer screening and blood pressure control.

## Methods

A retrospective cohort study was used to compare two groups. The intervention group were continuously enrolled individuals followed for 12 months (participating 2 + times in CFL for 3 months of the preceding 12 months). The threshold of 2 + meetings was set as a significant threshold for engagement for two reasons. This was the requirement from the partner plan for a member to be categorized as engaged and it was the number of events deemed necessary by the CFL team from their previous operational experience to begin to form trust amongst the members which was a key part of the intervention. The control group were matched controls (with at least 12 months of prior health plan membership but not enrolled in CFL) followed over the same 12-month period. Member matching was performed in R using the MatchIT package with a 0.25 caliper. A 1:1 tight caliper matching was used as it introduced less bias [20]. Propensity matching was done across age, sex, comorbidities, baseline utilization patterns and baseline claims. A difference of differences comparing pre- and post-exposure values between the two groups for outcomes of interest was calculated.

Study participants were Medicaid members from the state of Michigan, enrolled in the same health plan for both exposure and control groups. All study members were African American, defined by self-reported records extracted from claims data. A total of 738 members were matched to the same number of controls for the period of May 2021 to April 2022. Enrollees in the exposure groups were CFL eligible plan members who were previously exposed to CFL. Exposure was defined as having attended two or more CFL events in the preceding 3 months. Controls were CFL eligible plan members who had never been contacted about nor attended a CFL event. All members in the exposure group were directly contacted by the CFL team using multimodal methods including text, phone and door knocking campaigns and invited to participate.

Data were abstracted from longitudinal claims data.

**Fig. 1:**
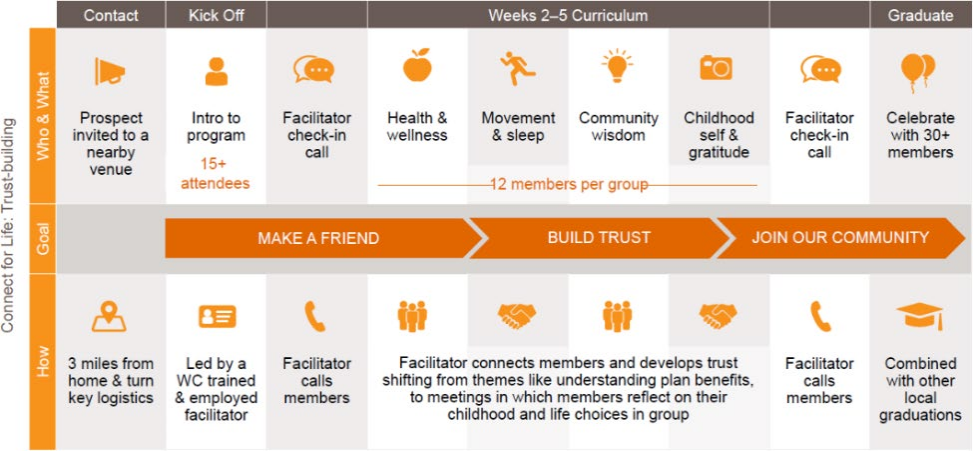
An overview of the Connect for Life community model Figure author Wider Circle who have granted full permission to the authors for reuse for purposes of publication

The CFL exposure involves multiple small groups of twelve enrollees participating in six weekly, in-person, hour-long meetings led by a culturally competent facilitator, who was sourced, trained, and managed by the CFL team. Members become part of a larger community after completing their weekly meetings in the smaller group setting. As a part of that larger community, they are invited to participate in health promotion meetings, group physical activities, and peer-to-peer local information exchanges about disease-specific wellness resources. Content across both settings includes understanding benefits, addressing housing needs, changing diet, tackling food insecurity, improving physical activity, practicing gratitude, and comprehending the importance of screening (see Fig. 1).

Outcomes of interest were related to healthcare utilization and cost. Inpatient stays were defined as the number of unique inpatient claims (wherein one claim ID relates to one admission) that occurred in the study timeframe per 1000 members. Inpatient paid amounts were defined as the total amount paid towards inpatient claims in USD over the study timeframe for that member (including lab and testing costs). ED Encounters were defined as the number of unique non-emergent [21] emergency department-related claims (wherein one claim ID relates to one ED event) that appeared in the given timeframe per 1000 members. The NYU algorithm was used to define non-emergent episodes of care [21]. ED Paid amounts were defined as the total amount paid towards non-emergent ED usage in USD (including lab and testing costs) for the given timeframe for that member. Office Visits were defined as the total number of unique claims (with a place-of-service code indicating the visit took place in an outpatient physician’s office wherein one claim ID relates to one office event) per 1000 members. Office Paid was defined as the total amount paid in USD towards office claims (including lab and testing costs) in the given timeframe for that member. Pharmacy claims were defined as the count of unique pharmaceutical claims that were paid over the study period. Pharmacy paid was defined as the total sum of all money spent on pharmaceutical claims over the study period. Per member, per month cost (PMPM) was defined as the average monthly cost of a member in USD, summing together all inpatient and ambulatory costs (including prescription pharmacy, lab, testing, and other costs) over the study period (Tables 1 and 2).

**Table 1:** Comparison of baseline characteristics between exposure group and control. Continuous variables: p-value for difference calculated from unpaired Welch’s T-test

Baseline characteristic	Exposure group	Control group	P value for difference between groups
Age, mean, range, years	42.2 (19–70)	42.0 (19–70)	0.724
Sex % female	58.1	58.1	1.00
Ethnicity % African American	100	100	1.00
Charlson comorbidity index, mean	0.505	0.505	1.00
Elixhauser comorbidity index	1.33	1.30	0.646
Inpatient utilization, mean per 1000	100	100	1.00
Non emergent ED utilization, mean per 1000	367	367	1.00
Outpatient utilization, mean per 1000	3423	3855	0.207
Per member per month cost (USD), mean	266	289	0.534
Inpatient paid (USD), mean	710	732	0.916
Non emergent ED paid (USD), mean	121	120	0.916
Outpatient paid (USD), mean	392	387	0.960
Prescription paid (USD), mean	920	1,284	0.232
Number of unique prescription claims in the pre-period	23.5	22.2	0.513

**Table 2:** Comparison of baseline characteristics of SDOH factors between exposure group and control using zip code derived averages and the ADI.

Baseline characteristic	Exposure group	Control group	Difference in value (exposure vs. control)
Employment rate (%)	84.7	87.2	-2.5
Population with poor English skills (%)	1.16	1.15	0.01
Median household income ($)	33,685	41,450	-7,765
Households receiving food stamp support (%)	36.8	30.0	6.8
No access to a vehicle (%)	21.3	17.0	4.3
Average distance to nearest urgent care (miles)	1.49	1.57	-0.08
Average distance to nearest emergency department (miles)	1.48	1.86	-0.38
Average ADI relative to the state	9.38	8.12	1.26
Households who rent (%)	54.4	44.9	10
Adults who did not complete high school (%)	16.7	14.2	2.5
Adults whose highest educational attainment is high school (%)	34.6	32.2	2.4

Secondary outcomes of interest were plan member retention and quality outcomes (as available from claims data), including Cervical Cancer Screening, Flu Vaccination, Breast Cancer Screening, Diabetes Eye Exam, Diabetes Nephrology Exam, Adult BMI Assessment, Adult Access to Preventative/Ambulatory Health Services (20 to 44 years old and 45 to 65 years old) and Controlling Blood Pressure (CBP) codified by standard definitions as used by the National Committee for Quality Assurance (NCQA) [22]. Cofounders included age, sex, gender, comorbidity scores (Charlson and Elixhauser), and zip code. Further comparisons on baseline SDOH were conducted using zip code level data available from AHRQ [23] and area deprivation index (ADI). For each group, the average of the members zip code-based SDOH rates were estimated. Due to the nature of this approach, both groups had very low variance due to the data being compiled at the zip code level. This meant there were a large number of results (members with the same zip code) that showed the same SDOH metric value and guaranteed an extremely strict confidence interval for any test for equality. As such, the decision was made to descriptively examine these versus conduct a test of equality which would be misleading. No outliers were excluded for linear variables or converted to categorical variables.

Member matching was performed in R using the MatchIT package with a 0.25 caliper and 1:1 matching yielding 738 member pairs. There were no individuals with missing data in the intervention group. Selection bias was mitigated through tight propensity matching of control participants with a 0.25 caliper to ensure they were as similar to the exposure group as possible. Recall bias was mitigated by verifying an individual’s exposure to CFL through independent records kept by the CFL operations team. The number of matched pairs was based on the number of members in the plan not enrolled in the exposure group that could be matched to those exposed while still meeting the caliper threshold [20].

The member retention analysis required a separate pool of candidates as the nature of the original analysis’ pre-post design ensured all candidates were in the pool after one year, (so member retention in the plan would always be 100% across both arms). To conduct this sub analysis the full list of CFL eligible members from April 2021 was pulled, and then marked as retained if they were still CFL eligible under the plan in April 2022. These cohorts were then separated by current CFL program status. The exposure group included people with the statuses ‘Enrolled Member’, ‘Inactive Member’, or ‘Discontinued Member’ who enrolled before April 2021. The control group contained everyone else Table 3.

**Table 3:** Comparison of exposure group and control for outcomes of interest. Continuous variables: p-value for difference calculated from unpaired Welch’s T-test. SD, standard deviation. All values given to 3 significant figures

Variable	Exposure Baseline	Exposure at 12 months	Change in exposure group	Control at Baseline	Control at 12 months	Change in control group	Difference between control and exposed	95% CI	P Value
Per Member per month costs (USD), Mean	266	352	86.0	289	490	201	-115	-210, -20.2	0.0175*
Inpatient paid amounts (USD), Mean	710	983	273	732	1703	971	-698	-1554, 158	0.110
Non emergent ED paid amounts (USD), Mean	121	124	3.22	120	197	77.0	-73.8	-164, 15.9	0.107
Outpatient paid amounts (USD), Mean	392	626	234	387	600	213	+ 21.5	-297, 340	0.895
Pharmacy paid amounts (USD), Mean	921	1410	489	1279	2046	767	-278	-693, 137	0.189
Inpatient utilization / 1000	100	149	48.8	100	180	79.9	-31.1	-94.1, 318	0.332
Non emergent ED utilization / 1000	367	407	39.3	367	466	98.9	-59.6	-162, 42.7	0.253
Outpatient utilization / 1000	3423	4669	1246	3855	5341	1486	-240	-910, 430	0.483
Flu Vaccine (%)	14.1	8.36	-5.71	15.3	9.04	-6.21	0.504	-3.23, 4.24	0.791
Adult BMI assessment (%)	74.2	72.5	-1.72	74.4	76.7	2.24	-3.96	-10.8, 2.85	0.254
Breast cancer screening (%)	37.7	39.6	1.89	44.0	46.0	2.00	0.113	-20.7, 20.4	0.991
Cervical cancer screening (%)	47.6	51.1	3.53	50.4	56.4	5.95	-2.42	-8.86, 4.02	0.482
Controlling blood pressure (%)	15.0	13.3	-1.77	11.6	12.6	1.05	-2.82	-14.8, 9.18	0.643
Diabetic retinopathy exam (%)	23.6	32.7	9.09	28.8	30.8	1.92	7.17	-14.6, 28.9	0.515
Diabetic kidney exam (%)	56.3	47.3	-9.06	63.5	57.7	-5.77	-3.29	-24.3, 17.6	0.754
Access to preventative health services (20–44 y/o) (%)	30.6	26.7	-3.93	40.2	27.9	-12.2	8.31	0.352, 16.3	0.041*
Access to preventative health services (45–64 y/o) (%)	40.5	43.5	3.06	40.4	46.1	5.67	-2.61	-12.6, 7.42	0.609
Hemoglobin A1c test (%)	20.0	23.6	3.64	9.62	17.3	7.69	-4.06	-24.3, 16.2	0.692

* p < 0.05

## Results

**Fig. 2:**
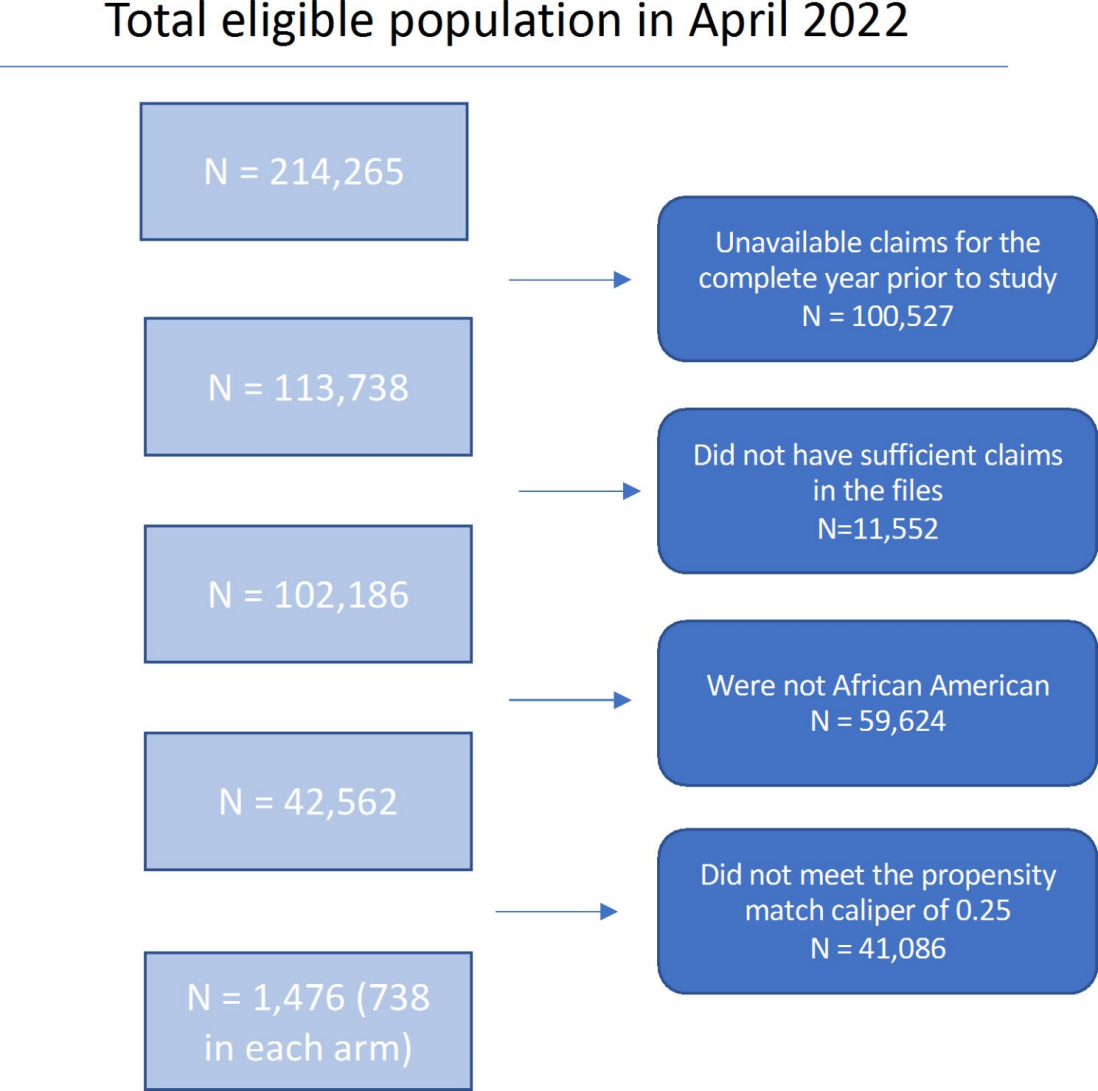
Flow diagram illustrating selection criteria that resulted in 738 matched pairs for final analysis between control and intervention

214,265 participants were eligible for CFL (and the study) based on available claims and location of the delivered intervention. 100,527 were removed from this cohort due to having unavailable claims for the complete year prior to the study leaving 113,738. 11,552 participants were removed for quality control as they did not have sufficient claims data in the files (wherein they did not have single medical claim attached to the patient ID for the preceding year). This left 102,186 individuals. 59,624 participants were removed because they were not African American leaving 42,562 individuals. A further 41,086 were removed through the process of optimally matching individuals to the exposure arm to create as tight a propensity match as possible [20] across baseline factors. This left a final N = 738 participants in the control group and 738 in the exposure group for a total of 1,476 participants (Fig. 2).

Across the exposure and control groups, age ranged from 19 to 70 and the mean in each group was 42.2 and 42.0 years respectively. 58.1% were female in the exposure group and 58.1% in the control group. Ethnicity was 100% African American across both groups. Similar paid amounts, number of distinct claims and utilization patterns at baseline were apparent across both exposure and control groups with no significant differences. There were no significant differences in Charlson or Elixhauser comorbidity index scores. Across SDOH factors there were no global trends between the two groups zip code-based baseline SDOH factors. It is worth noting the exposure group did have lower household income and a higher percentage of economically related items such as higher rates of food-stamp utilization, rent and rates of no access to a vehicle.

After one year, the CFL exposure was correlated with a significantly lower per member per month costs in the African American population by USD $115 (95% CI $20.2 to $210). All other utilization and actuarial measures moved in an improved direction in the exposure group, but results were non-significant. Inpatient utilization and non-emergent emergency department utilization showed trends of decreased rates of 31/1000 and 60/1000 respectively. The pattern of utilization trended favorably towards lower acuity settings. Mean inpatient paid amounts showed trends of decreasing by $698 per claim. Mean non-emergent Emergency Department paid amounts showed trends of decreasing by $74 per claim. Mean Outpatient paid amounts showed trends of increasing by $22 per claim. Mean pharmacy paid amounts showed trends of decreasing by $278 Table 4.

**Table 4:** Comparison of exposure group and control for member retention. Number of individuals in each group represented as a count. P-value for difference calculated from unpaired Welch’s T-test

Variable	Exposure Baseline	Exposure at 12 months	Change in exposure group	Control at Baseline	Control at 12 months	Change in control group	Difference between control and exposure	95% CI and P value
Member retention (%)	1834 (count)	1636 (count)	89.2% retention	129,320 (count)	111,018 (count)	85.9% retention	3.62%	< 0.01 (1.92, 4.79)

A significant increase in the access to preventive health services quality metric was observed of 8.31% (95% CI 0.352–16.3) for the 20–44 year-old metric on exposure to CFL. All other quality metrics did not show significant differences. The exposure group performed slightly better in percentage of flu vaccination, breast cancer screening and diabetic retinopathy exams completed and slightly worse in percentage of cervical cancer screening, BMI assessment, HBA1c and diabetic kidney exams completed. There was no global trend of improving or worsening quality between exposed and control groups.

Member retention in the plan for those members exposed to CFL was significantly higher in the exposure group vs. the control group by 3.62%.

Overall, the results of the study show significant reduction in costs and beneficial trends in utilization while maintaining or improving quality. This indicates that the exposure may be an effective tool for improving value.

## Discussion

Compared to controls, peer support recipients of CFL generated significantly lower per member per month costs ($115, (95% CI $20.2 to $210)). The PMPM calculation included all prescription, lab, and testing costs. Non-significant trends for lower utilization of acute care settings were also observed on exposure to CFL. There was no difference in clinical quality across any quality metrics between the two groups except for access to preventive health services in 20–44 year olds, which improved by 8.31% on exposure to CFL.

The study suggests that a community-based exposure that addresses health behaviors and SDOH may be effective in the African American population and reduce total cost of care. In addition, there is some evidence that the exposure may favorably improve utilization patterns – including inpatient and non-emergent emergency department utilization -- albeit non-significantly. Finally, the exposure is not associated with worsening quality metrics and may improve some such as access to preventative health services. This is consistent with existing literature that affirms the positive effects of community-based wellness programs and adds to a growing body of literature that interventions that address health behaviors and SDOH lower cost and improve utilization patterns [24].

## Limitations

As this is a retrospective cohort study, selection bias may have been present if individuals who left the plan and had missing claims data over the year of follow up were systematically different from those studied. Additionally, individuals who were committed to the program through attending at least two events and were categorized as ‘exposed’ would also be exposed to a form of selection bias as they could be more likely to take health-benefiting actions, versus those who didn’t. This would explain the improvement observed [25]. Efforts to address this included creating as tight a propensity match as possible to reduce selection bias while still retaining sufficient study participants to adequately power the study. Factors that went beyond demographics, baseline utilization, health outcomes and cost such as employment, education level, literacy, vehicle ownership and ADI were included and compared between exposure and control and no large differences were found. As the pre-exposure period occurred during the COVID-19 pandemic, this may have attenuated all preferential utilization pattern findings, as preventative health utilization was globally depressed throughout this period [26]. The effect on results would have been to mask significant findings in preferential utilization, thus understating the true effect.

## Conclusion

Our study found that leveraging community and “taking the plan to them,” through a community-based program that addresses health and SDOH, may be an effective strategy for changing health behaviors. Future studies include a dose response analysis would be helpful to understand the effects of each additional attendance and whether the intervention behaves in a linear or multiplicative manner along with an intention to treat analysis that prospectively captures important elements of SES, and assesses people referred to the program with actual participation as an outcome. Health plans and risk-bearing providers could look to contract with existing community-based wellness services to better manage risk and retain their populations, especially for members who are at risk for healthcare disparities.

## Electronic supplementary material

Below is the link to the electronic supplementary material.

Supplementary Material 1

## Data Availability

Study used retrospective anonymized insurance claims with no patient identifiable information.
