# Supplementary material for: Improving value for underserved populations with a community-based intervention: a retrospective cohort study
Source: Arch Public Health. 2023 May 29;81:96. doi: 10.1186/s13690-023-01117-z (PMC10225756; doi:10.1186/s13690-023-01117-z)
Supplement: Supplementary file 1 — Supplementary Material 1 [file 13690_2023_1117_MOESM1_ESM.docx]

**Aim and Scope Statement.**

To whom it may concern,

The aim of this study was to evaluate the effectiveness of a community-based peer intervention “connect for life” in addressing cost, quality and addressing inequity for underserved communities when delivered in the form of a supplemental benefit in partnership with health plans. The intervention specifically tackles Social Determinants of Health.

**What is already known on this topic** – Health inequity and SDOH are well known to influence and drive low value care. It is unclear how effective supplemental benefits leveraging community to address these factors are, and how they differ for different ethnicities.

**What this study adds** – This study shows that an upstream community intervention may be an effective benefit to address inequity and SDOH for African American populations and lower costs whilst improving or maintain quality.

**How this study might affect research, practice, or policy**- Health payers, whether national or privatized, could look to contract with existing community-based wellness services to better manage risk and retain their populations, improve member experience and address concerns commonly captured in STARS ratings, especially for members who are prone to healthcare disparities.
